# The Care of the Disabled Soldier

**Published:** 1917-03-10

**Authors:** 


### The Hospital

The Workers' Newspaper of Administrative Medicine and Institutional Life, Administration, National Insurance and Health.

No. 1605, Vol. LXI. SATURDAY, MARCH 10, 1917. PRICE ONE PENNY.

### THE CARE OF THE DISABLED SOLDIER.

The soldier who has been maimed or crippled in the service of his country is one of the many serious problems provided by the war, and, as the witness of our streets testifies, the problem is an increasing one. What proportions it will reach by the time the war comes to an end the future alone can declare. In the very nature of things the numbers of such men must increase, and it may be doubted whether a scheme adequate to meet the situation has yet been devised. The responsibility, it is clear, must be shouldered by the Government. The demand is not for charity, and it cannot be left to any voluntary philanthropic organisation. The men have suffered in the cause which the nation has declared its own, and the nation through its official machinery must discharge what is a plain obligation of honour. Equally manifest is the condition that a mere provision of pensions for disabled men is an unwise and unsatisfactory solution of the question. Such a method meets neither the case of the men nor the case of the nation. The men in the great majority of cases have many years of life in front of them, and to leave them in crippled idleness is to condemn them to a future without hope and to the risk of moral disaster. Further, it is certain that in many instances these men under suitable training may be fitted for work which will qualify them to earn their own livelihood. They are therefore among the potential workers of the nation, and thus economic considerations not less than the claims of honour prompt to a determination to appreciate and to meet their position. There is still another reason why any scheme to be effective must enjoy an official status. So long as these men are in the Army they are soldiers, and therefore subject to discipline and control. Whatever reasonable methods of treatment may be selected may be imposed upon them by military order, and habit and the influence of authority will incline them to obey. But a very different state of matters arises as soon as such men are discharged from the Army. Institutions and organisations may be at their service, but who shall compel them to utilise these opportunities? The wise and far-seeing among them will do so no doubt, but such qualities must not be presumed in the majority of young men recently released from military discipline and having a certain though modest pension in their pocket. The need for this official retention of control is all the more essential seeing that the physical treatment and re-education needed to fit most of these men for efficient civil life occupy a considerable period of time and must be pursued systematically and continuously. An interrupted and irregular attendance in such training is merely a waste of both time and money. It is therefore in the interests of the men themselves, as well as to secure the fruitful use of public money, that the curative methods available should be applied under military authority until, in each instance, the maximum benefit to be expected has actually been obtained.

The principles we have just stated govern the situation when this is viewed, as it ought to be viewed, from a generous and national standpoint. When we say that the Army regards the question from a different angle we have not the least intention of suggesting an unpleasant contrast. The business of the Army is with soldiers, or with men who may be made into soldiers. If a soldier is for the time being ill or injured the supreme military question, so far as he individually is concerned, is—Can he again be made fit for service? If this is possible, curative methods are within the Army schedule, for they are part of the scheme by which the fighting ranks can be maintained and strengthened. But when injuries are of such a degree or nature as permanently to render the sufferer useless for military service, treatment necessarily lacks a military motive. It may be quite right that these unfortunate men should be cared for by some appropriate organisation, but the Army is not this organisation. The military career of the men is closed beyond hope and they have no place in the fighting machine. Such is the not altogether unnatural argument which may be advanced by those responsible for military administration, and we take it that, consciously or unconsciously, it lies at the bottom of the reported decision that every man who is certified as permanently unfit must be discharged from the Army within three weeks of the issue of such a certificate. For reasons given in the earlier part of this article we regard this decision as unjust to the men and unfair to the nation, and we trust that an expression of public opinion will bring it to nought. The nation is deeply committed to the men who have suffered in its service, and it will not permit technical hindrances to prejudice the treatment of these men in the best possible conditions. The military administration must accept the national will; they cannot act autocratically in this matter. An offer to treat maimed and disabled men as out-patients at the nearest military hospital is wholly inadequate to the position, and the public conscience, we are assured, will never endorse so feeble and futile a proposal.

Our position, in a word, is that both justice and national economy demand for these men the systematic and skilful application of the educational and therapeutic measures best calculated to secure for each individual a maximum of civil efficiency. This end, we feel sure, will not be attained if the men are cast loose from the discipline and control to which they have been accustomed. What is needed is that under the play of these influences physical therapeutic methods and the education and training of the workshop shall be so applied as to confer on each man his maximum value alike to himself and to the country for which he has suffered. In this way will be combined justice to the individual and economic service to the country.

